# Tuning Gas Sensor Selectivity via Morphological Engineering of Au Catalysts on TiO_2_‐based Nanosheets

**DOI:** 10.1002/smll.202509219

**Published:** 2025-10-22

**Authors:** Sol Han, Yong Whan Kim, Sang Hyun Ji, Seung Yong Lee, Eunki Yoon, Aelim Ha, Soohyung Park, Hyung‐Seok Kim, Dong Won Chun, Myung Sik Choi, Changhyun Jin, Kyu Hyoung Lee, Jeong Yun Hwang

**Affiliations:** ^1^ Department of Materials Science and Engineering Yonsei University Seoul 03722 Republic of Korea; ^2^ Advanced Analysis Center Korea Institute of Science and Technology (KIST) Seoul 02792 Republic of Korea; ^3^ Energy Storage Research Center Korea Institute of Science and Technology (KIST) Seoul 02792 Republic of Korea; ^4^ Department of Materials Science and Engineering Pohang University of Science and Technology (POSTECH) Pohang 37673 Republic of Korea; ^5^ Department of Nano & Advanced Materials Science and Engineering Kyungpook National University Sangju 37224 Republic of Korea

**Keywords:** Au morphology, molecular weight‐dependent sensing, selectivity, surface energy engineering, TiO_2_ nanosheets

## Abstract

Recent studies on catalyst development have primarily focused on discovering novel catalyst compositions, rather than exploiting the morphology and interface effects of existing catalytic materials. This study develops a novel material‐driven gas sensor selectivity strategy by tailoring the surface energy of TiO_2‐_ *_y_* (TO) and Ti_1‐_ *_x_* Pt*_x_* O_2‐_ *_y_* (TPO) nanosheet (NS) supports to control the morphology of decorated Au catalysts. Through precise modulation of the substrate surface energies of TO/TPO NSs, the sizes and shapes of the Au catalysts can be engineered from nanoparticles (NPs) to micro‐sized sheets without significant changes in chemical composition. This morphological control enables the realization of molecular weight‐dependent gas selectivity, achieving enhanced detection of target gases, such as CO_2_, ethanol, benzene, and toluene, with molecular weights of 40 or higher. In contrast to conventional approaches reliant on passive material properties, this strategy leverages engineered material interfaces and catalyst morphologies to actively direct gas sensing behavior. Furthermore, these results overturn the conventional notion that smaller catalysts necessarily yield higher reactivity, demonstrating that catalyst size and shape, governed by substrate‐material interactions, can be key determinants of chemical reactivity. This finding provides a new pathway for the rational design of selective gas sensors through advanced materials engineering.

## Graphical abstract

It is shown that Au morphology, rather than size alone, governs gas selectivity when supported on TO and TPO nanosheets. Pt substitution releases internal energy to increase surface energy, stabilizing extended Au sheets. This morphology‐driven pathway enables selective detection of heavier gases and challenges the long‐standing paradigm that “smaller is always better.”

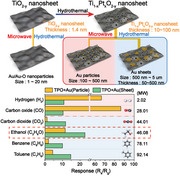

## 1 Introduction

A catalyst does not directly participate in a chemical reaction but instead modifies the activation energy barrier, thereby accelerating or decelerating the reaction rate.^[^ ^1^, ^2^, ^3^ ^]^ It has long been accepted that nanometer‐scale catalysts are inherently advantageous for such reactions, as their large surface‐to‐volume ratio increases the probability of interaction with reactant molecules. Accordingly, noble metals such as Au, Ag, Pt, and Pd have been extensively employed in metal oxide semiconductor (MOS)‐based gas sensors to enhance their response to various target gases, including oxidizing gases (O_2_, NO, NO_2_, N_2_O, SO_2_), reducing gases (H_2_, CO, NH_3_, H_2_S, CH_4_), volatile organic compounds (VOCs; HCHO (formaldehyde), C_2_H_2_ (acetylene), C_2_H_6_O (ethanol), C_3_H_6_O (acetone), C_6_H_6_ (benzene), C_6_H_10_ (cyclohexene), C_7_H_8_ (toluene)), and H_2_O.^[^ ^4^, ^5^, ^6^ ^]^ However, even when using the identical MOS materials and catalysts, gas sensor selectivity often varies unpredictably between light and heavy gas molecules, and there is currently no established framework to rationalize or predict this behavior. For example, studies employing Pt‐functionalized SnO_2_, one of the most widely used gas sensing materials, have reported markedly different selectivity profiles, favoring H_2_, NH_3_, CO, or ethanol depending on subtle variations in experimental conditions.^[^ ^7^, ^8^, ^9^, ^10^ ^]^ As a result, selectivity in gas sensing remains largely empirical, with no consistent trends identifiable across the published literature.

Historically, most gas sensor research has predominantly focused on optimizing three performance metrics, response magnitude, response time, and recovery time (the so‐called “3R” factors), which are relatively straightforward to measure and interpret.^[^ ^11^, ^12^, ^13^, ^14^, ^15^ ^]^ Variables such as operating temperature, gas concentration, humidity, reproducibility, and long‐term stability are typically evaluated in terms of their influence on 3R performance. In contrast, selectivity has traditionally been approached in a passive manner, relying heavily on experimental trial‐and‐error. To date, most explanations for gas selectivity have centered on chemical interactions, including the following: (1) electron affinity effects at the semiconductor/gas interface,^[^ ^16^, ^17^ ^]^ (2) the formation of new chemical species,^[^ ^18^, ^19^ ^]^ and (3) the bond dissociation energies of gas molecules.^[^ ^20^, ^21^ ^]^ Numerous studies have reported noble metal‐decorated oxides with enhanced sensitivity. However, these works have primarily focused on achieving improved responses to specific target gases without addressing the underlying origin of selectivity. The influence of physical attributes such as catalyst size, shape, and dimensionality on gas sensing behavior has been largely overlooked. Recent catalyst development efforts have primarily focused on discovering novel catalyst compositions, such as precious‐metal‐free alternatives, eco‐friendly formulations, or materials with enhanced durability and low‐temperature performance, rather than exploiting the morphology and interface effects of existing catalytic materials.^[^ ^22^, ^23^, ^24^ ^]^

In this study, we present a fundamentally new approach to achieving selective gas sensing by engineering the surface energy of TO and TPO NS supports to precisely control the morphology of supported Au catalysts. By systematically tuning the size and shape of Au, ranging from nanoparticles to micro‐sized sheets, we demonstrate that gas selectivity can be actively modulated according to the molecular weight of the target gas. In particular, we showed that micro‐sized Au sheets exhibit superior selectivity toward gases with molecular weights ≥ 40 (e.g., CO_2_, C_2_H_6_O, C_6_H_6_, and C_7_H_8_) than Au particles, thereby challenging the conventional assumption that smaller catalysts inherently confer higher reactivity. Furthermore, we confirmed that both 3R performance and selectivity can be systematically enhanced through this morphology‐based strategy, offering new insights into the role of physical catalyst parameters in the rational design of a selective gas sensor. This perspective moves beyond the conventional paradigm that “smaller catalysts are always more active” and provides a new framework to rationalize and design selective gas sensors.

## 2 Results and Discussion

**Figure 1:**
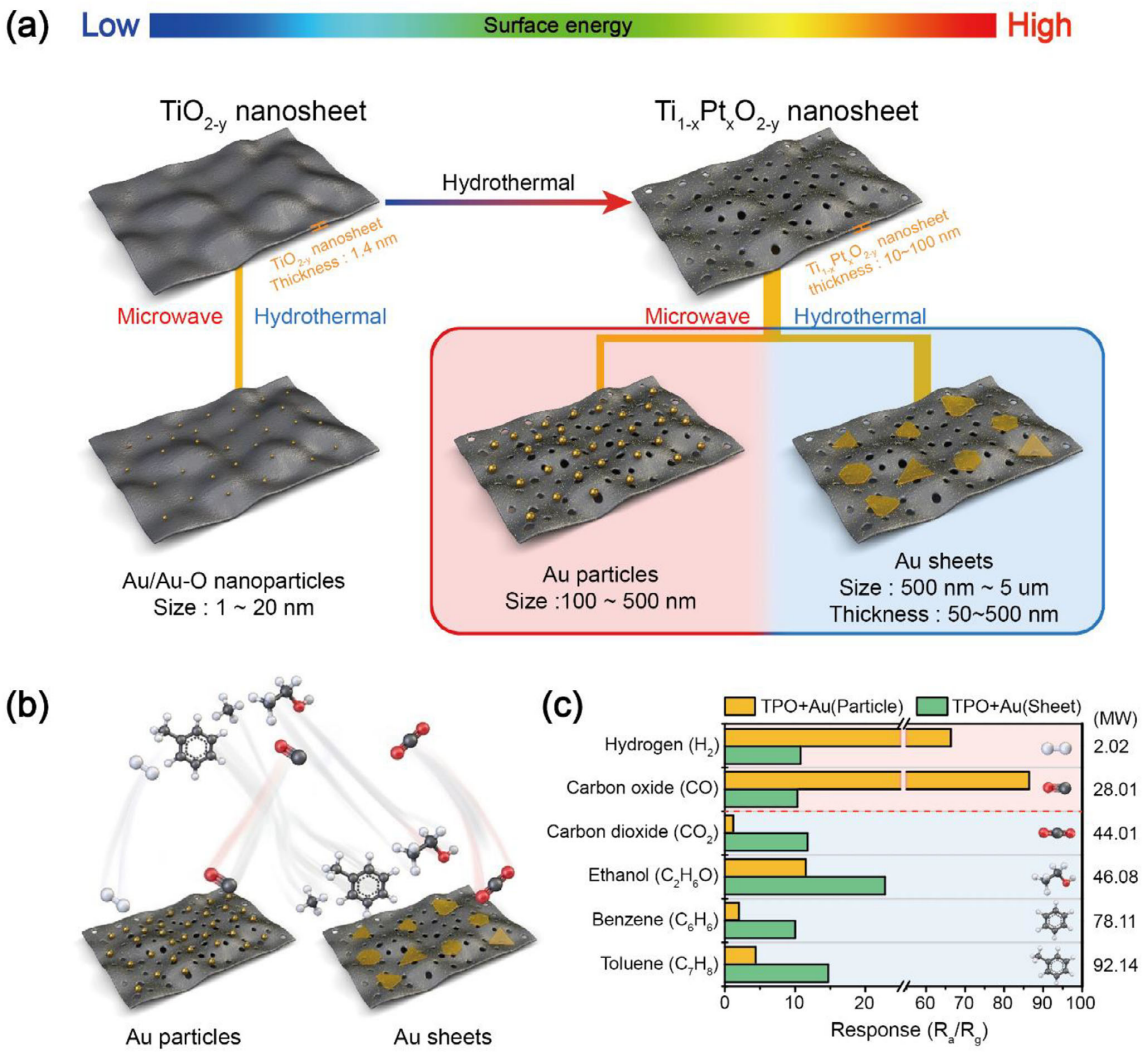
a) Schematic illustration of the morphological evolution of Au catalysts on TiO_2‐y_ (TO) and Ti_1‐x_Pt_x_O_2‐y_ (TPO) nanosheets (NSs) as a function of surface energy and deposition method. b) Conceptual visualization of gas‐catalyst interactions between target gas molecules and Au catalysts of different morphologies (particles vs sheets) on TPO. c) Comparative gas sensing responses toward H_2_, CO, CO_2_, C_2_H_6_O, C_6_H_6_, and C_7_H_8_ for TPO NSs decorated with Au particles and sheets.

To clearly illustrate the concept of gas selectivity in this study, we compared two types of NSs (TO and TPO NSs) with significantly different surface energies, along with their corresponding Au‐decorated forms: TO NSs decorated with Au nanoparticles (NPs, 1 – 10 nm), TPO NSs decorated with Au particles (100 – 500 nm), and TPO NSs decorated with Au sheets (500 nm – 5 µm in width and 50 – 500 nm in thickness). Au decoration was carried out using either a microwave or hydrothermal method (**Figure** **1**). As shown in Figure 1a, when Au catalysts were deposited on TO NSs via either Au or Au‐O NPs with sizes less than 10 nm were uniformly distributed across the surface, consistent with previous reports.^[^ ^25^, ^26^ ^]^ In contrast, Au decoration on TPO NSs yielded markedly different results. The microwave method produced large spherical Au particles ranging from 100 to 500 nm with no detectable Au─O species. In the hydrothermal approach, triangular or hexagonal Au sheets ranging from 500 nm to 5 µm were formed. These Au structures existed solely in metallic form, without any oxide phases. This morphological variation is attributed to the increased surface energy from TO to TPO NS; as surface energy increases, Au atoms tend to aggregate rather than disperse, forming larger, distinct structures such as spheres or sheets.^[^ ^27^, ^28^, ^29^ ^]^ As shown in Figure 1b, these structural differences significantly affect the sensor's response to target gases, depending on their molecular weights. For light gases with molecular weights ≈40 or less (e.g., H_2_ and CO), stronger responses were observed when Au existed as sub‐500 nm particles (≈2.02 for H_2_ and ≈28.01 for CO). In contrast, for heavier gases such as CO_2_, C_2_H_6_O, C_6_H_6_, and C_7_H_8_, significantly improved responses were achieved when Au was present as micron‐sized sheets (≈44.01 for CO_2_, ≈46.08 for C_2_H_6_O, ≈78.11 for C_6_H_6_, and ≈92.14 for C_7_H_8_), as shown in Figure 1c. This finding contrasts with the conventional understanding that smaller catalysts typically offer higher reactivity due to their larger surface‐to‐volume ratios.^[^ ^30^, ^31^, ^32^ ^]^ Our results suggest that nanoscale Au catalysts may be insufficient to effectively activate and interact with larger gas molecules. Instead, the use of larger, well‐defined Au sheets provides a more favourable surface for detecting these heavier gases.

**Figure 2:**
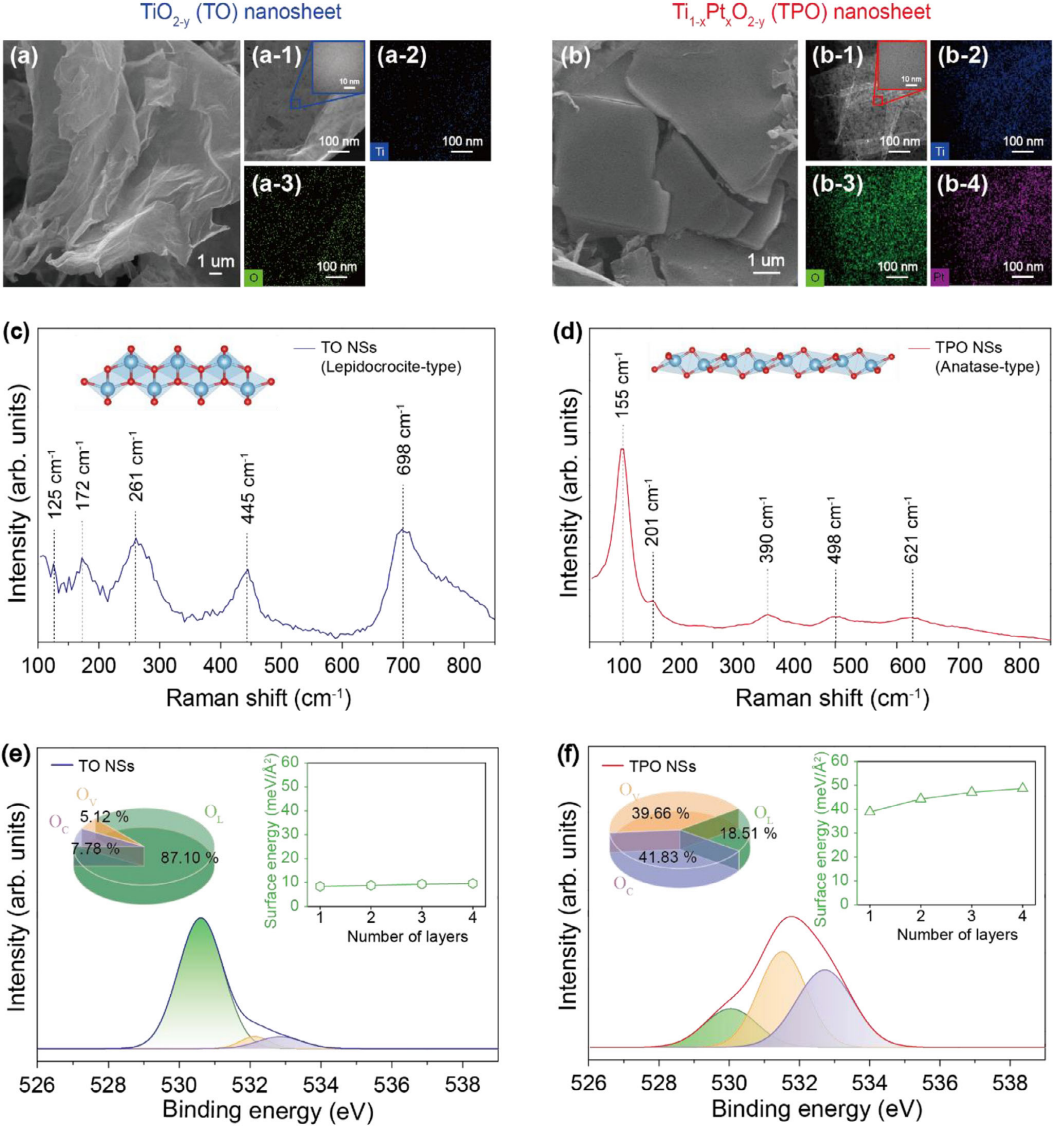
SEM images of a) TO and b) TPO NSs. TEM and EDS elemental mapping images of (a‐1 to a‐3) TO and b‐1 to b‐4) TPO NSs. Raman spectra of c) TO NSs (lepidocrocite‐type) and d) TPO NSs (anatase‐type). Deconvoluted XPS O 1s spectra and the corresponding thickness‐dependent surface energy (right axis) for e) TO nanosheets and f) TPO NSs.

Detailed insights into the distinct surface energy states of the materials are presented in **Figure** **2**. As shown in Figure 2a, the TO NSs exhibit a uniform distribution of Ti and O across the sheet. In contrast, the TPO NSs (Figures 2b and S2, Supporting Information) show a significantly higher density of uniformly distributed surface defects, likely resulting from the incorporation of Pt. Raman spectroscopy further confirms the structural differences between TO and TPO NSs (Figure 2c, d). The TO NSs exhibit characteristic peaks at 125, 172, 261, 445, and 698 cm^−1^, corresponding to a lepidocrocite‐type single‐crystalline structure. A Ti‐O‐Ti stretching mode, consistent with KTLO (the known precursor to TO NSs) is also observed (Figure S3, Supporting Information).^[^ ^33^ ^]^ In TPO, where partial substitution of Ti by Pt occurs, the structure transitions to an anatase‐type phase, as indicated by Raman peaks at 155, 201, 390, 498, and 621 cm^−1^.^[^ ^34^ ^]^ OM images (Figure S4, Supporting Information) reveal morphological changes consistent with the increased surface energy, while XRD analysis (Figure S5, Supporting Information) confirms the presence of sheet‐like anatase structures.^[^ ^35^ ^]^ This structural transformation is accompanied by increased Ti‐O bond disruption and a higher concentration of oxygen vacancy (O_V_), as evidenced by XPS analysis (Figure S6, Supporting Information).^[^ ^36^ ^]^ XPS O 1s spectra combined with surface energy calculations provide further insight into the TO–TPO transition. Figure 2e shows the O 1s peak of TO nanosheets, where lattice oxygen (O_L_) is dominant at 87.10% (530.6 eV), with minor contributions from oxygen vacancy (O_V_, 5.12%, 532.1 eV) and chemisorbed oxygen (O_C_, 7.78%, 533.3 eV).^[^ ^37^ ^]^ The inset illustrates the layer‐dependent surface energy of TO, which remains relatively low and stable (e.g., 8.35 meV Å^−^^2^ for a monolayer, gradually increasing to 9.57 meV Å^−^^2^ by four layers). Raman data further confirm that TO and TPO nanosheets possess distinct crystal structures. In the case of TO (lepidocrocite structure), the nanosheets remain in a relatively unstable state with excess internal energy. Upon Pt substitution, this energy is released to the exterior, leading to the formation of pores and a substantial increase in oxygen vacancies on the surface. These changes collectively result in a significant rise in surface energy. As a consequence, the structure stabilizes into the anatase phase, and the redistribution of energy to the surface is in excellent agreement with our DFT simulations. Figure 2f presents the O 1s spectrum of TPO nanosheets. In contrast to TO, O_L_ decreases sharply to 18.51% (530.1 eV), while O_V_ and O_C_ rise significantly to 39.66% (531.5 eV) and 41.83% (532.7 eV), respectively. The inset shows that the surface energy of TPO is consistently higher than that of TO and increases more steeply with layer number (from 38.90 meV Å^−^^2^ at one layer to 48.68 meV Å^−^^2^ at four layers). Supporting this observation, gas sensing experiments conducted at room temperature with 20 ppm ethanol revealed a higher response from TPO NSs (2.3) compared to TO NSs (1.2) (Figure S7a, Supporting Information). Moreover, TPO NSs demonstrated markedly faster response and recovery times (68 s and 139 s, respectively) compared to TO NSs (194 and 195 s) (Figure S7b, Supporting Information). Beyond ethanol, TPO NSs also showed superior sensitivity to other gases, including H_2_, CO, CO_2_, C_6_H_6_, and C_7_H_8_ (Figure S7c, Supporting Information), further validating the impact of structural and surface energy modifications on gas sensing performance.

**Figure 3:**
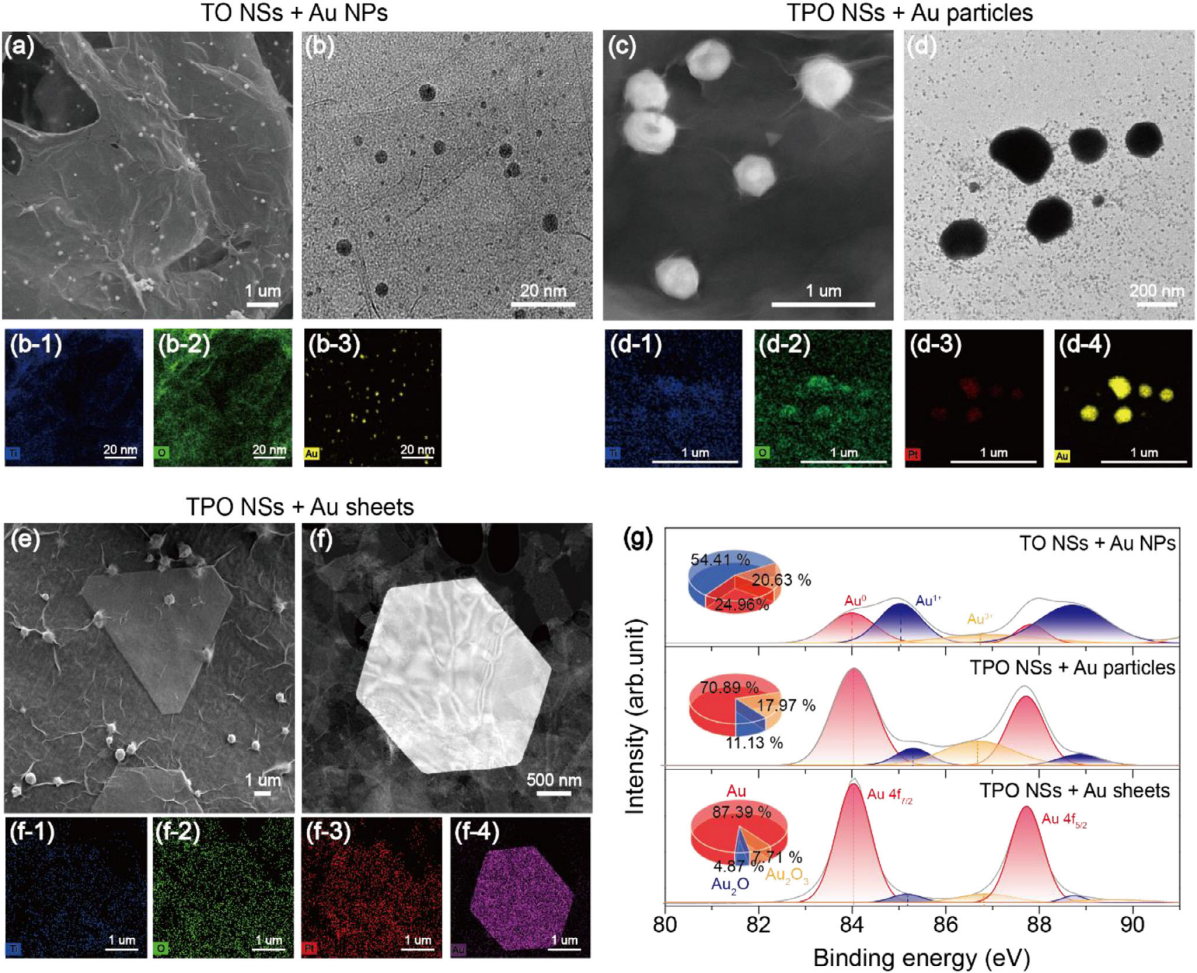
a,b) TEM images of TO NSs decorated with Au NPs. (b‐1 to b‐3) EDS elemental mapping confirms the uniform distribution of Ti, O, and Au. c,d) TEM images of TPO NSs decorated with Au particles. (d‐1 to d‐4) EDS elemental mapping shows the co‐presence of Ti, O, Pt, and Au. e,f) TEM images of TPO NSs decorated with Au sheets. (f‐1 to f‐4) EDS elemental mapping reveals the co‐presence of Ti, O, Pt, and Au. g) XPS analysis of Au 4f spectra showing chemical state distributions (Au^0^, Au^+^, and Au^3+^) for TO decorated with (top) Au NPs, TPO decorated with (middle) Au particles, and (bottom) Au sheets.

The influence of surface energy on the deposition behavior of Au catalysts on TO and TPO NSs is illustrated in **Figures** **3** and S8 (Supporting Information). As shown in Figure 1, hydrothermal treatment of TO NSs with an Au precursor results in the uniform decoration of Au NPs several nanometers in size, as confirmed by elemental mapping (Figure 3a,b). In contrast, the morphology of Au deposited on TPO NSs varies significantly depending on the synthesis method. Under microwave‐assisted synthesis, large Au particles are formed. Elemental mapping reveals the co‐localization of Ti, O, Pt, and Au, with the Au particles appearing coarse and aggregated, particularly near Pt regions (Figure 3c,d). Conversely, hydrothermal synthesis produces 2D Au sheets in triangular, truncated triangular, or hexagonal shapes, which are distributed across the TPO NSs surface at micron‐scale dimensions (Figures 3e,f and S9a–d, Supporting Information). XRD analysis (Figure S9e, Supporting Information) confirms that these Au structures are compositionally and spatially distinct from Ti, O, and Pt. To investigate the chemical states of Au, XPS analysis was conducted on Au‐decorated TO and TPO NSs as shown in Figure 3g. For TO NSs decorated with Au NPs, the relative proportions of Au^0^, Au^+^, and Au^3+^ were 24.96% (84.06 eV), 54.41% (85.03 eV), and 20.63% (86.86 eV), respectively. In TPO NSs decorated with Au particles, these values shifted to 70.89% (84.12 eV), 11.13% (85.38 eV), and 17.97% (86.77 eV), while TPO NSs decorated with Au sheets exhibited 87.39% (84.01 eV), 4.87% (85.16 eV), and 7.71% (86.85 eV), respectively.^[^ ^38^ ^]^ These results confirm that the enhanced surface energy of the host material drives Au to be decorated either in sheet or particle form. Correspondingly, the XPS Au 4f spectra (Figure 3g) demonstrate that Au on TPO exists predominantly in its elemental state compared to TO, thereby experimentally verifying its superior catalytic activity. These results indicate that as the surface energy increases during the transition from TO to TPO NSs, Au catalysts tend to exist predominantly in its metallic state rather than in oxidized forms. This metallic stabilization is more pronounced under hydrothermal synthesis than microwave‐assisted synthesis. Notably, the hydrothermal method significantly reduces the presence of Au_2_O and Au_2_O_3_, highlighting that the oxidation state of Au can be tuned by controlling surface energy. This provides a strategic route for tailoring catalyst properties for specific applications through surface energy modulation.

**Figure 4:**
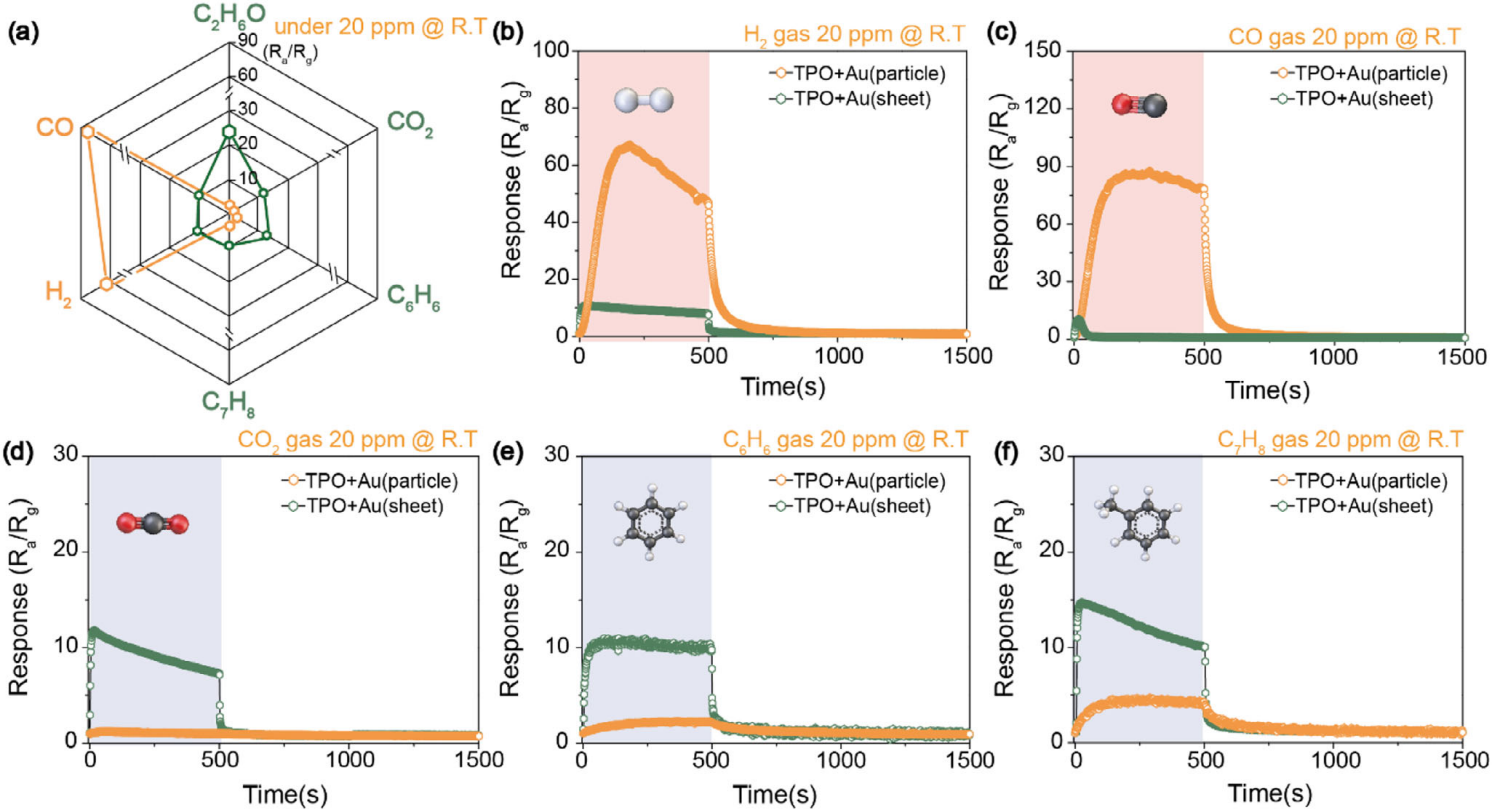
a) Radar plot comparing the gas sensing responses of TPO NSs decorated with Au particles and sheets toward six target analytes (20 ppm each) at room temperature. Comparison of the responses for individual gas species: b) H_2_, c) CO, d) CO_2_, e) C_6_H_6_, and f) C_7_H_8_.

**Figure 5:**
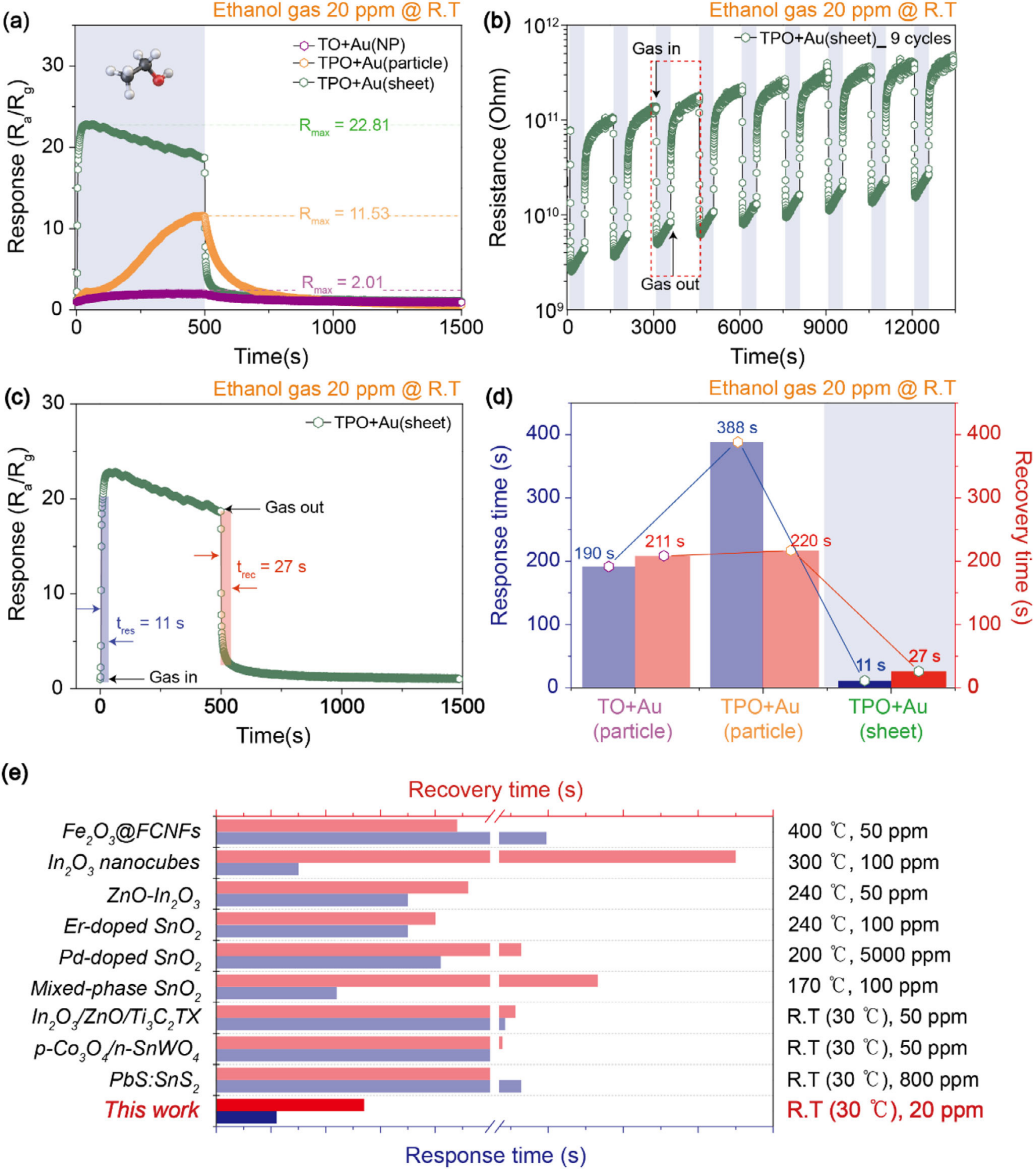
a) Comparison of responses of TO NSs decorated with Au NPs and TPO NSs decorated with Au particles and sheets at 20 ppm ethanol concentration at room temperature. b) Repeatability and c) response and recovery times of TPO NSs decorated with Au sheets at 20 ppm ethanol concentration at room temperature. d) Comparison of response and recovery times of TO NSs decorated with Au NPs and TPO NSs decorated with Au particles and sheets at 20 ppm ethanol concentration at room temperature. e) Summary of response and recovery times in other studies on ethanol gas sensing in different environments.

To evaluate the surface reactivity of TO and TPO NSs modified with various Au catalysts, gas sensing measurements were conducted using six target analytes (H_2_, CO, CO_2_, C_2_H_6_O, C_6_H_6_, and C_7_H_8_) as shown in the gas sensing setup (Figure S10, Supporting Information). As shown in **Figures** **4**a and S11 and S12 (Supporting Information), all analytes produced markedly higher responses on TPO NS‐based sensors compared to TO NS‐based counterparts. Specifically, for low molecular weight gases such as H_2_ and CO (Figure 4b,c), the highest responses were observed with TPO NSs decorated with Au particles. In contrast, for heavier gases such as CO_2_, C_6_H_6_, C_7_H_8_ (Figure 4d–f), and C_2_H_6_O (**Figure** **5**a), the TPO NSs decorated with Au sheets exhibited superior sensing performance. These results reveal a consistent and novel trend. For analytes with molecular weights ≤ 40, sub‐micron spherical Au catalysts are more effective, whereas for analytes with molecular weights > 40, 2D Au sheets provide significantly better sensing responses. These finding highlights that molecular‐weight‐dependent selectivity originates from morphology‐driven catalytic interactions. Furthermore, it confirms that the observed selectivity is not a simple function of Au loading, but instead reflects a fundamental mechanism whereby catalyst morphology dictates the sensing pathway. This indicates that gas reactivity is not solely determined by surface area or catalytic activity, but is strongly influenced by the dimension and size of the Au catalyst. Traditionally, noble metal catalysts in gas sensors enhance performance through mechanisms such as the spill‐over effect, where gas molecules interact with adsorbed oxygen species, triggering charge transfer and altering sensor resistance via mobile carrier generation.^[^ ^39^, ^40^, ^41^ ^]^ This mechanism, however, does not account for the physical morphology of the catalyst, as it primarily involves the pre‐contact activation of gases, rather than interactions at the material surface. Thus, the actual reaction site (whether at the Au‐gas interface, semiconductor‐gas interface, or the Au‐semiconductor‐gas triple junction) must be carefully distinguished. For larger gas molecules, it is more plausible that sensing predominantly occurs at the ternary interface (Au‐semiconductor‐gas).^[^ ^42^ ^]^ This provides a rationale for why larger or 2D Au structures, despite their lower surface‐to‐volume ratios, outperform NPs in detecting high molecular weight analytes. In such cases, 1D contact lines formed by extended Au sheets may enable more effective and stable interactions with bulky gas molecules than the 0D point contacts typical of NPs. These findings highlight the importance of catalyst morphology in designing gas sensors. If gas response depends on the nature of the interfacial contact, conventional NP‐based catalyst designs may be inherently limited for certain analytes, particularly those with higher molecular weights. Tailoring the dimensionality of the catalyst interface thus offers a promising strategy for enhancing selectivity and sensitivity in gas sensing applications. Compared with the particle‐based TO NSs decorated with Au NPs (Figure S13, Supporting Information), Figures 5 and S13 (Supporting Information) present the comprehensive sensing characteristics of the ethanol sensor, which exhibited the highest selectivity, based on the sheet‐like TPO NSs decorated with Au sheets. At room temperature and 20 ppm ethanol, the response values were 22.81 for TPO NSs decorated with Au sheets, 11.53 for TPO NSs decorated with Au particles, and 2.01 for TO NSs decorated with Au NPs (Figure 5a). Furthermore, across nine repeated measurement cycles, the response variation remained within 1%, confirming excellent reproducibility and operational stability (Figure 5b). The sensor also demonstrated rapid response (11 s) and recovery (27 s) times (Figure 5c), outperforming other Au morphologies (Figure 5d) and previously reported ethanol sensors (Figure 5e).^[^ ^43^, ^44^, ^45^, ^46^, ^47^, ^48^, ^49^, ^50^, ^51^ ^]^ Consistent performance was maintained across ethanol concentrations of 20, 10, 6, 2, and 1 ppm, yielding a calculated limit of detection (LOD) of 0.99 ppm (Figure S14, Supporting Information). However, performance degradation was observed under humid conditions (Figure S15, Supporting Information) and in cases involving Au desorption (Figures S16 and S17, Supporting Information). This limitation is consistent with earlier reports (Table S1, Supporting Information),^[^ ^52^, ^53^, ^54^, ^55^, ^56^, ^57^, ^58^, ^59^, ^60^, ^61^ ^]^ highlighting that maintaining Au in a stable micro‐scale sheet morphology is critical for reliable ethanol sensing. To address this challenge, potential strategies such as defect‐engineered anchoring of Au, hydrophobic surface functionalization, or protective passivation layers could be adopted to suppress Au detachment and mitigate humidity effects. Incorporating these approaches will be an important direction for translating our morphology‐driven sensing concept into practical devices. On the other hand, the optimal Au doping concentration was determined not by the total Au content but by the ability to form and maintain sheet‐like morphologies. Too little Au failed to create continuous sheets, while excessive Au led to aggregation and collapse, weakening gas selectivity. Control experiments (Figures S16 and S17, Supporting Information) further confirmed that the enhanced response arises specifically from the preserved sheet morphology. Thus, the chosen Au concentration reflects a balance, sufficient to induce stable sheet formation while avoiding collapse. It is particularly noteworthy that most MOS sensors struggle to achieve reliable 3R (response‐recovery‐repeatability) performance at room temperature due to insufficient carrier activation.^[^ ^62^, ^63^ ^]^ By contrast, our system overcomes this limitation, offering robust room‐temperature sensing. This work introduces and validates a novel gas sensing mechanism based on 2D, sheet‐like Au catalysts, which are especially effective for detecting large molecular weight analytes.

## 3 Conclusion

In this study, we demonstrated that gas sensor selectivity can be precisely tuned by modulating the morphology of Au catalysts through surface energy engineering of TiO_2_‐based nanosheet (NS) supports. Specifically, TiO_2‐y_ (TO) and Ti_1‐x_Pt_x_O_2‐y_ (TPO) NSs facilitated the formation of distinct Au morphologies from nanoparticles (NPs) to micro‐sized sheets depending on their surface energy and the synthesis method employed. While TO supported uniform decoration of Au NPs, the higher surface energy of TPO enabled the formation of larger Au particles or well‐defined 2D Au sheets. Crucially, this morphological variation was found to have a profound impact on gas selectivity, closely correlated with the molecular weight of the target analyte. For low molecular weight gases (≤40), such as H_2_ and CO, a sensor based on TPO NSs decorated with Au particles exhibited superior performance. Conversely, for higher molecular weight gases (> 40), including CO_2_, C_2_H_6_O, C_6_H_6_, and C_7_H_8_, superior sensing performance was achieved with TPO NSs decorated with sheet‐like Au structures. These findings challenge the conventional notion that smaller catalysts inherently offer higher reactivity due to greater surface area. Instead, we show that larger catalyst morphologies can facilitate more effective interaction with bulky gas molecules, likely through improved physical interface matching or enhanced contact geometry. This work establishes a new design paradigm for gas sensors: by tailoring the surface energy of the support to control catalyst morphology, selective gas detection can be achieved through morphology‐driven mechanisms rather than relying solely on chemical affinity. This approach offers a robust and tunable strategy for the rational development of high‐performance, molecule‐specific gas sensors, particularly well‐suited for room‐temperature applications. Moreover, this morphology‐driven strategy provides a versatile platform for fabricating low‐power, selective gas sensors compatible with next‐generation wearable electronics.

## 4 Experimental Section

### Chemicals

Potassium(I) carbonate (K_2_CO_3_, 99%), lithium(I) carbonate (Li_2_CO_3_, 99.99%), titanium(IV) oxide (TiO_2_, rutile, 99.99%), and molybdenum(VI) oxide (MoO_3_, 99.98%) were purchased from Kojundo Korea Co., Ltd. Hydrochloric acid (HCl, 1.0 N standardized solution) was obtained from Thermo Scientific. Tetrabutylammonium hydroxide solution (TBAOH, 40 wt.% in H_2_O) and 2‐propanol (IPA, ≥99.5%) were supplied by Sigma‐Aldrich. Hydrogen tetrachloroaurate(III) hydrate (HAuCl_4_∙4H_2_O) and hydrogen hexachloroplatinate(IV) hydrate (H_2_PtCl_6_∙6H_2_O) were sourced from Kojima Chemicals Co., Ltd. All chemicals were used as received without further purification.

### Preparation of TO NSs

TiO_2‐y_ nanosheets (TO NSs) were synthesized via physical and chemical exfoliation using a cation exchange process. The precursor material, lepidocrocite‐type layered titanate (K_0.8_Ti_1.73_Li_0.27_O_4_, KTLO), was synthesized using a flux method, following a previously reported procedure.^[^ ^25^ ^]^ Subsequently, 4.5 g of KTLO was stirred in 300 mL of 0.5 m HCl at 80 rpm for 5 days to form H_1.07_Ti_1.73_O_4_∙H_2_O (HTO), enabling ion exchange of interlayer K^+^ with H_3_O^+^ within the TiO_6_ octahedral layers. Next, 1.2 g of the resulting HTO was added to a mixture of 10.26 mL TBAOH solution and 300 mL deionized (DI) water, followed by stirring at 150 rpm for 2 weeks to achieve organic molecules intercalation. The final TO NSs colloid was obtained by centrifugation (6000 rpm, 10 min), followed by dialysis against DI water using a membrane for 3 days (Figure S1, Supporting Information).

### Preparation of TO NSs decorated with Au NPs

Gold nanoparticles (Au NPs) were decorated onto TO NSs via a one‐step hydrothermal method. Specifically, 0.23 g of HAuCl_4_∙4H_2_O was dissolved in 10 g of 2‐propanol under stirring at room temperature to form a transparent solution. The Au precursor solution was then mixed with the TO NSs colloid at a mass ratio of 1:4. The resulting mixture was transferred to a 100 mL Teflon‐lined autoclave and heated at 70 °C for 4 h (Figure S1, Supporting Information).

### Preparation of TPO NSs

Ti_1‐x_Pt_x_O_2‐y_ nanosheets (TPO NSs), in which Ti was partially substituted with Pt, were synthesized via a hydrothermal method. Specifically, 0.15 g of H_2_PtCl_6_∙6H_2_O was dissolved in a solvent mixture of 0.18 g of 2‐propanol and 2.82 g of DI water under stirring at room temperature to form a transparent solution. The Pt solution was then mixed with the TO NSs colloid at a mass ratio of 1:4. The resulting mixture was transferred to a 100 mL Teflon‐lined autoclave and heated at 70 °C for 4 h (Figure S1, Supporting Information).

### Preparation of TPO NSs Decorated with Au Particles and Sheets

Au‐decorated TPO NSs with either particle or sheet morphology were synthesized via microwave and hydrothermal methods, respectively. First, 0.23 g of HAuCl_4_∙4H_2_O was dissolved in 10 g of 2‐propanol under stirring at room temperature to prepare a transparent Au precursor solution. This solution was mixed with the TPO NSs dispersion at a mass ratio of 1:4. For the formation of Au particles, the mixture was transferred to a 100 mL microwave‐safe vial and irradiated at 1000 W for 1 min using a microwave reactor, resulting in TPO NSs decorated with Au particles (Figure S1, Supporting Information). For the synthesis of Au sheets, the same mixture was transferred to a 100 mL Teflon‐lined autoclave and subjected to hydrothermal treatment at 70 °C for 4 h, yielding TPO NSs decorated with Au sheets (Figure S1, Supporting Information).

### Characterization

X‐ray Diffraction (XRD) patterns were collected using an Ultima IV diffractometer (Rigaku) equipped with a Cu Kα radiation source (*λ* = 0.15 406 nm), operated at 40 kV and 40 mA. The crystal structures of TO and TPO NSs were analyzed over a 2*θ* range of 5 – 55°, while Au‐based phases were characterized from 10° to 90°. Morphological characterization was conducted using scanning electron microscopy (SEM, JEOL JSM‐7800F) and optical microscopy (OM, Olympus BX53M), and transmission electron microscopy (TEM, JEOL JEM‐F200). Elemental composition was analyzed via energy‐dispersive X‐ray spectroscopy (EDS) attached to the TEM. X‐ray photoelectron spectroscopy (XPS) measurements were performed using a Thermo Fisher Scientific Nexsa system with a monochromatic Al Kα source (1486.6 eV). Raman spectroscopy (XPER RF, Nanobase) with a 532 nm laser was employed to investigate phase transitions and chemical bonding configurations.

### Measurement of Gas Sensing Properties

Gold electrodes were sputter‐coated in a finger‐type pattern onto a SiO_2_‐coated substrate, which served as the platform for sensor fabrication. The sensing material was subsequently deposited onto the electrode‐patterned substrate via spray coating. The assembled gas sensors were placed inside a sealed gas chamber housed within a temperature‐controlled box furnace. Gas sensing performance was evaluated at room temperature under various target gas concentrations (1, 2, 6, 10, and 20 ppm). The total gas flow rate was maintained at 500 standard cubic centimeters per minute (sccm) using mass flow controllers, with dry air serving as the carrier gas. Electrical resistance values in air (*R*_a_) and under exposure to target gases (*R*_g_) were recorded, and the sensor response was defined as *R* = *R*_a_/*R*_g_. The target analytes included H_2_, CO, CO_2_, ethanol (C_2_H_6_O), benzene (C_6_H_6_), and toluene (C_7_H_8_). Response time and recovery time were defined as the time required to reach 90% of the total resistance change upon gas injection and subsequent removal, respectively. To investigate humidity effects, ethanol sensing tests were conducted under both dry and humid environments (relative humidity = 50%, 30 °C).

## Conflict of Interest

The authors declare no conflict of interest.

## Supporting information



Supporting Information

## Data Availability

The data that support the findings of this study are available from the corresponding author upon reasonable request.
