# Supplementary material for: Tuning Gas Sensor Selectivity via Morphological Engineering of Au Catalysts on TiO2‐based Nanosheets
Source: Small. 2025 Oct 22;21(48):e09219. doi: 10.1002/smll.202509219 (PMC12674107; doi:10.1002/smll.202509219)
Supplement: Supplementary file 1 — Supporting Information [file SMLL-21-e09219-s001.docx]

**Supplementary Information**

**Tuning Gas Sensor Selectivity via Morphological Engineering of Au Catalysts on TiO_2_-based Nanosheets**

*Sol Han, Yong Whan Kim, Sang Hyun Ji, Seung Yong Lee, Eunki Yoon, Aelim Ha, Soohyung Park, Hyung-Seok Kim, Dong Won Chun, Myung Sik Choi*, Changhyun Jin*, Kyu Hyoung Lee*, Jeong Yun Hwang**

S. Han, Y.W. Kim, S.H. Ji, S.Y. Lee, E. Yoon, A. Ha, H.S. Kim, C. Jin, K.H. Lee, J.Y. Hwang

Department of Materials Science and Engineering

Yonsei University

Seoul 03722, Republic of Korea

E-mail: z8015026@yonsei.ac.kr (C. Jin)

E-mail: khlee2018@yonsei.ac.kr (K.H. Lee)

E-mail: jyhawng@yonsei.ac.kr (J.Y. Hwang)

E. Yoon, A. Ha, S. Park

Advanced Analysis Center

Korea Institute of Science and Technology (KIST)

Seoul 02792, Republic of Korea

H.S. Kim

Energy Storage Research Center

Korea Institute of Science and Technology (KIST)

Seoul 02792, Republic of Korea

D.W. Chun

Department of Materials Science and Engineering

Pohang University of Science and Technology (POSTECH)

Pohang 37673, Republic of Korea

M.S. Choi

Department of Nano & Advanced Materials Science and Engineering
Kyungpook National University

Sangju 37224, Republic of Korea

E-mail: ms.choi@knu.ac.kr

**Table S1.** Examples of gas sensing with one-to-one correspondence between large catalysts and large molecular weight gases

| **Materials** | **Sensing gas** | **Material size** | **Ref.** |
| --- | --- | --- | --- |
| Nanostructured SnO_2_ | Ethanol | 1.2-1.5 μm particles | **[52]** |
| Ordered porous ZnO nanosheets | Ethanol | Submicron sheets | **[53]** |
| SnO_2_@Bi_2_O_3_ Core-Shell Heterojunction Structure | Ethanol | 1-10 μm particles | **[54]** |
| G-modified ZnO coral-like nanosheets | Ethanol | Submicron sheets | **[55]** |
| Porous LaFeO_3_ microspheres | Ethanol | >3 μm particles | **[56]** |
| Porous, 3D-hierarchical α-NiMoO_4_ rectangular nanosheets | Ethanol | 1-10 μm particles | **[57]** |
| Ordered Mesoporous SnO Micro Sheets | Ethanol | 1-10 μm particles | **[58]** |
| Zinc oxide nanonets with hierarchical crystalline nodes | Ethanol | 1-10 μm particles | **[59]** |
| MOF-Derived Single-Atom Sn-Doped ZnO Nanosheet | Acetone | 500 nm-1 μm NSs | **[60]** |
| Hollow rambutan-like zinc ferrite | Toluene | Submicron particles | **[61]** |


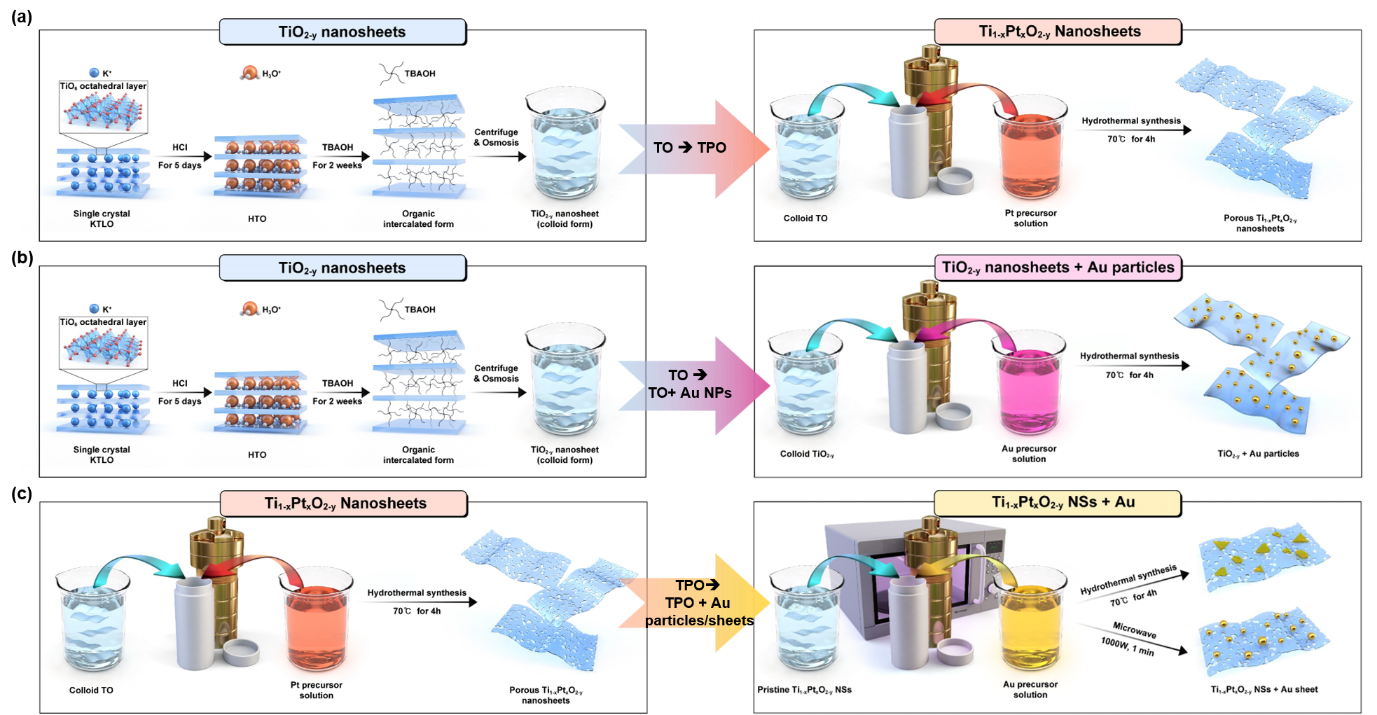


**Figure S1**. Schematic illustration of the synthesis methods of (a) TO and TPO NSs, (b) TO NSs decorated with Au NPs, and (c) TPO NSs decorated with Au particles and sheets.


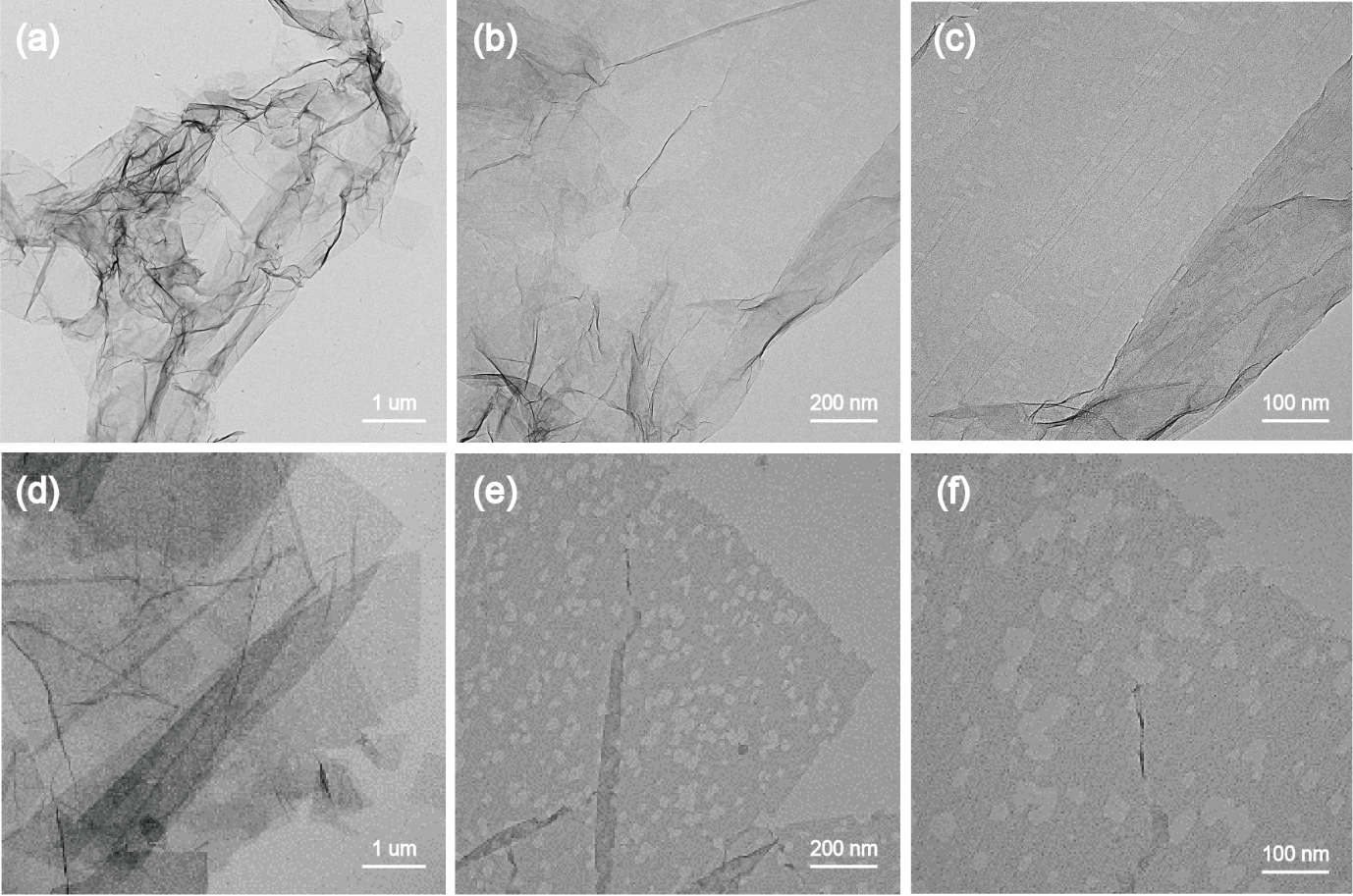


**Figure S2**. Representative TEM images of (a-c) TO and (d-f) TPO NSs.


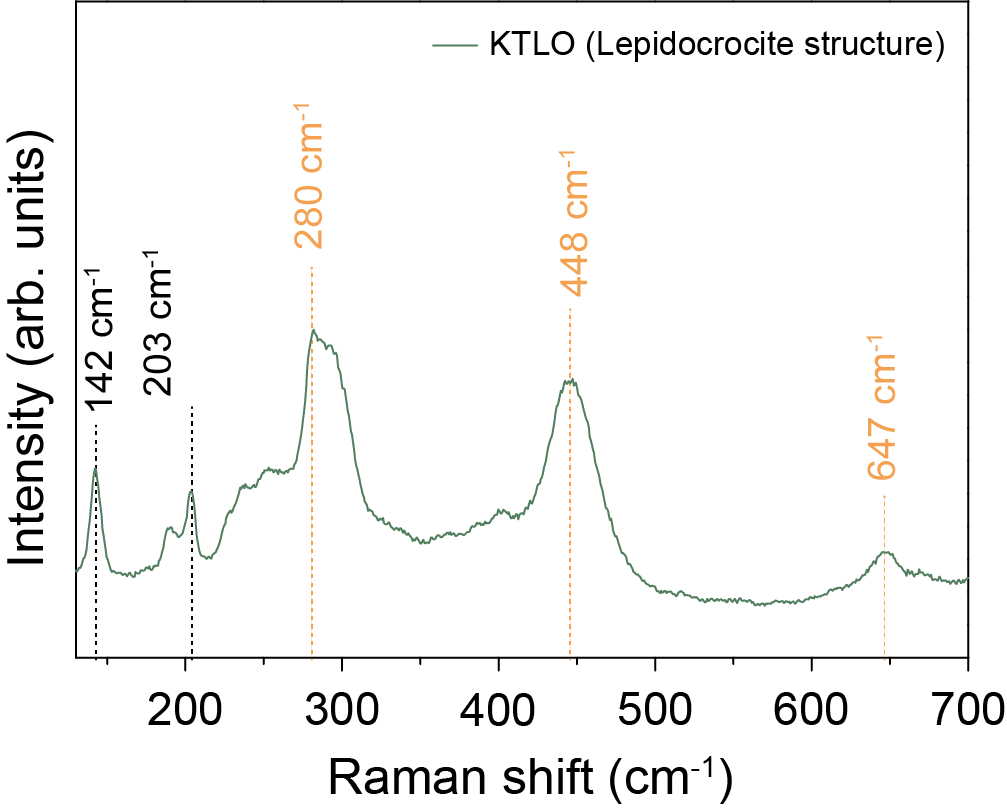


**Figure S3**. Raman spectrum of KTLO.


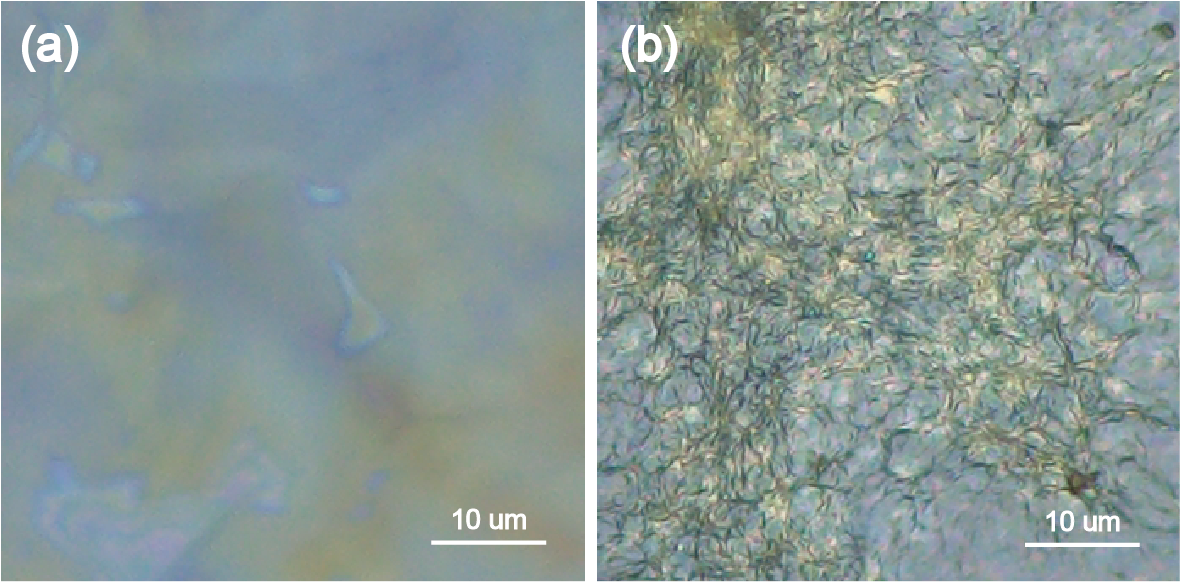


**Figure S4**. OM images of (a) TO and (b) TPO NSs.


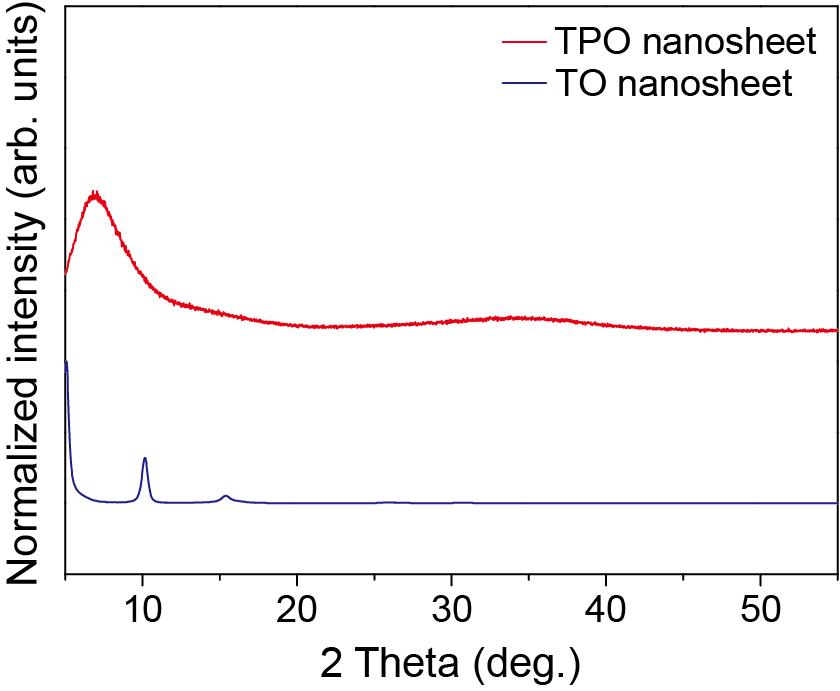


**Figure S5**. XRD patterns of TO and TPO NSs.


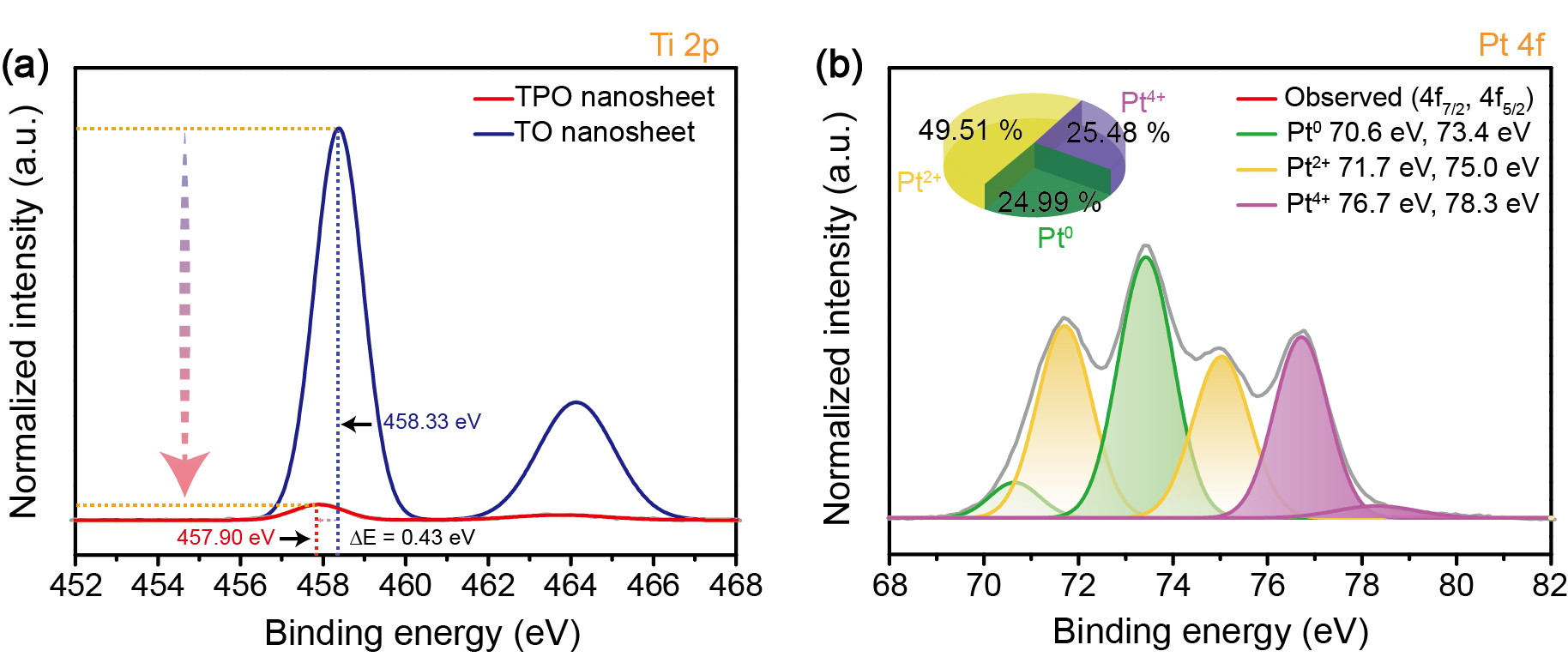


**Figure S6**. XPS spectra comparing the chemical bonding of TO and TPO NSs. (a) Ti 2p core-level spectra and (b) Pt 4f spectra deconvoluted into multiple oxidation states (Pt^0^, Pt^2+^, and Pt^4+^) with their corresponding percentages.


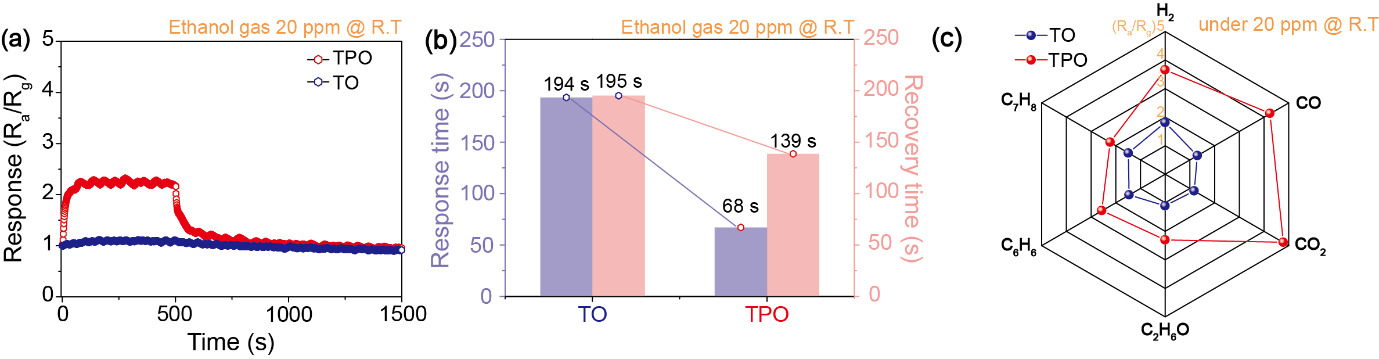


**Figure S7**. Comparison of (a) response and (b) response and recovery times of TO and TPO NSs for 20 ppm ethanol gas at room temperature. (c) Radar plot comparing gas selectivity of TO and TPO NSs.


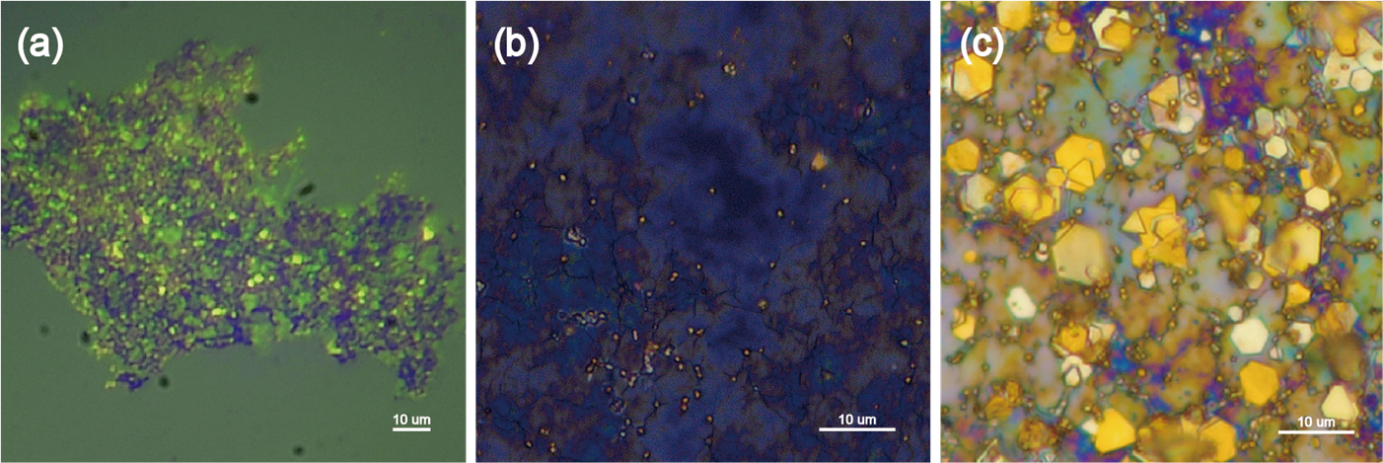


**Figure S8**. OM images of (a) TO NSs, (b) TPO NSs decorated with Au particles and (c) TPO NSs decorated with Au sheets.


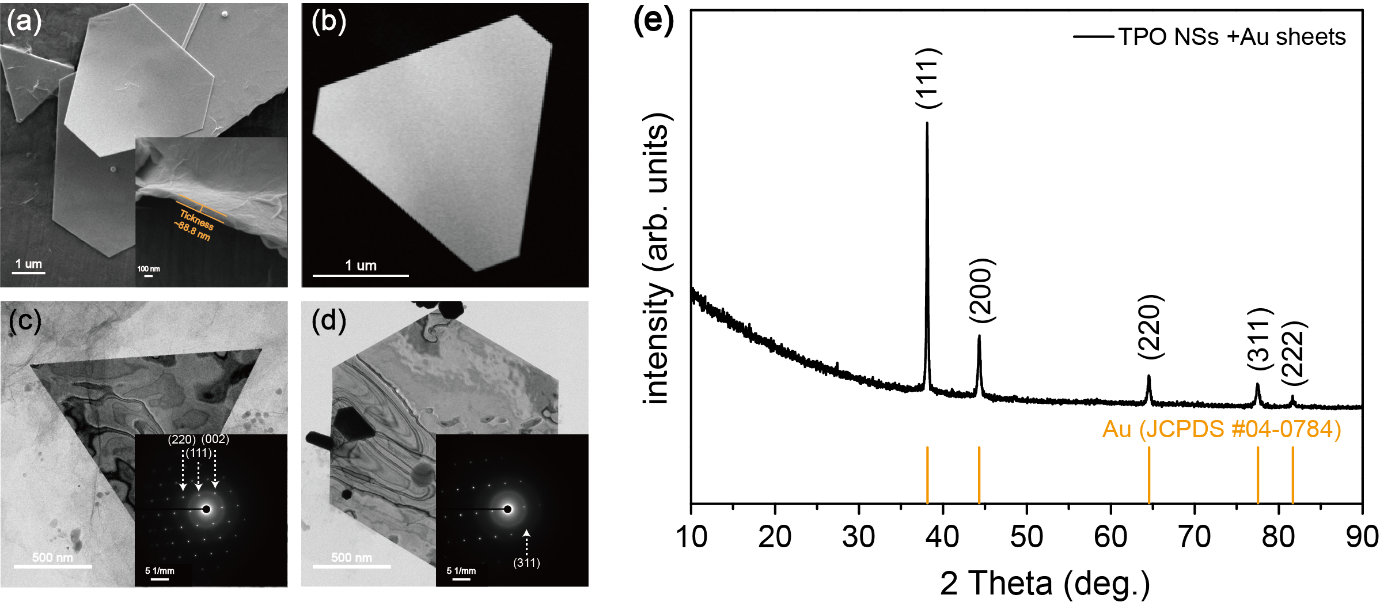


**Figure S9**. Structural and crystallographic analysis of TPO NSs decorated with Au sheets. (a-d) SEM and TEM images showing well-defined Au sheets on TPO NSs. (e) XRD pattern of TPO NSs decorated with Au sheets.


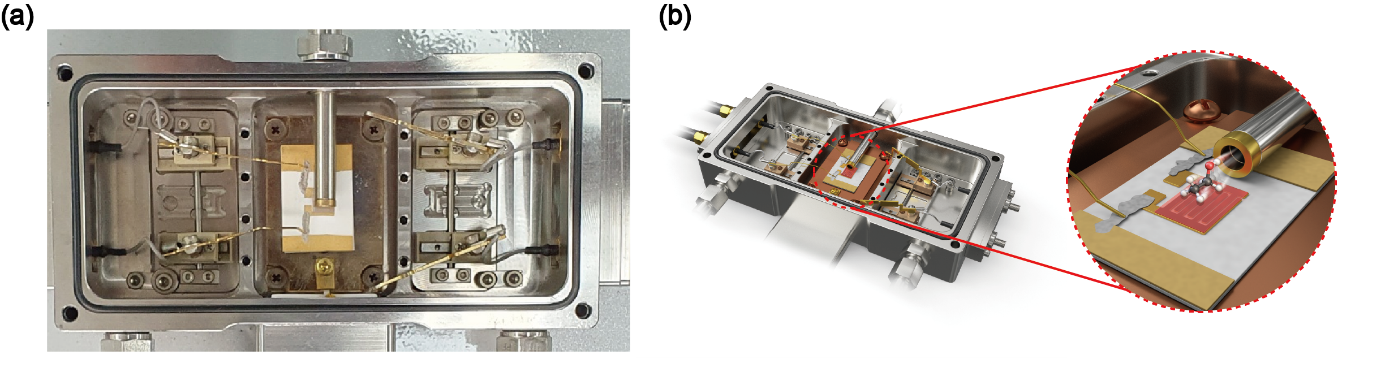


**Figure S10**. (a) Photograph and (b) schematic of the gas sensing measurement setup.


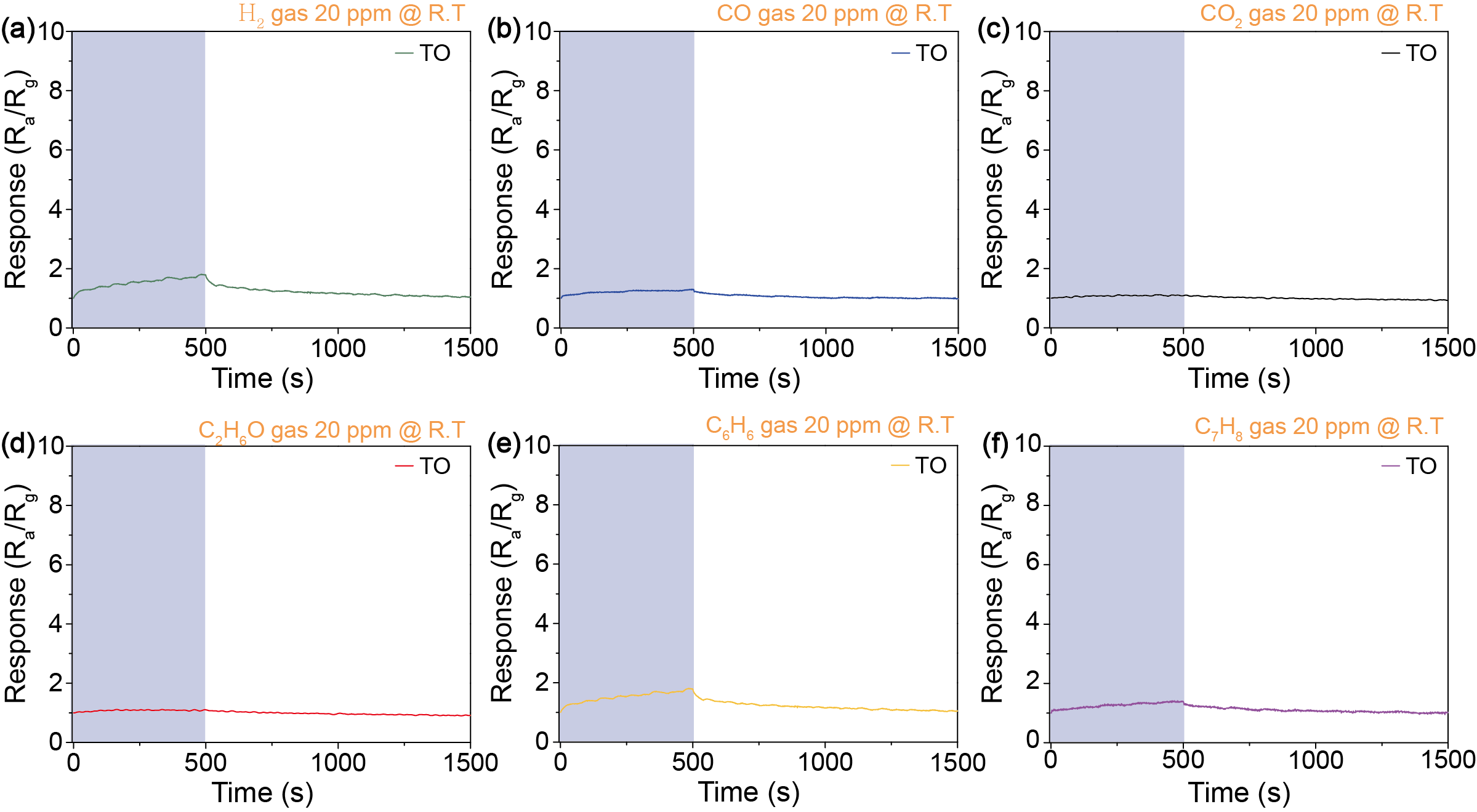


**Figure S11**. Response curves of TO NSs-based sensor to various target analytes (20 ppm each) at room temperature. (a) H_2_, (b) CO, (c) CO_2_, (d) C_2_H_6_O, (e) C_6_H_6_, and (f) C_7_H_8_ gases.


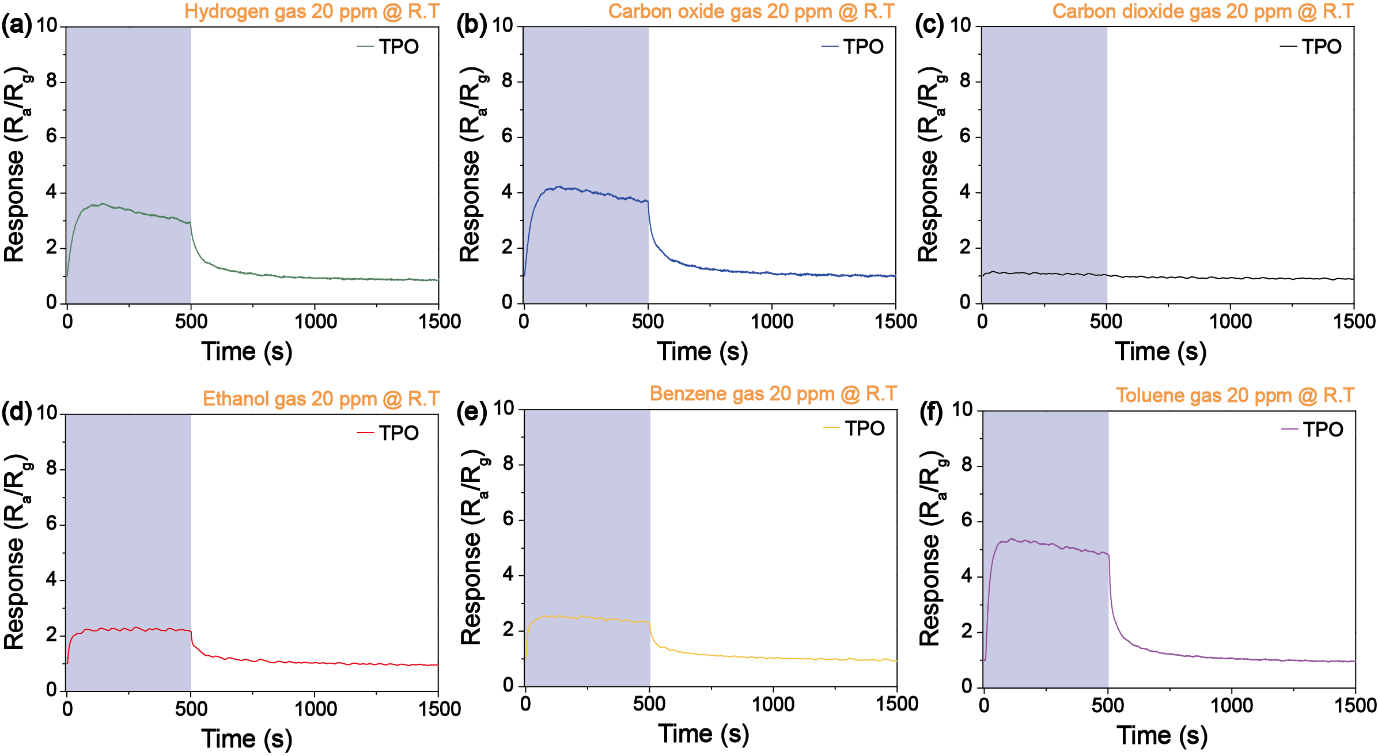


**Figure S12**. Response curves of TPO NSs-based sensor to various target analytes (20 ppm each) at room temperature. (a) H_2_, (b) CO, (c) CO_2_, (d) C_2_H_6_O, (e) C_6_H_6_, and (f) C_7_H_8_ gases.


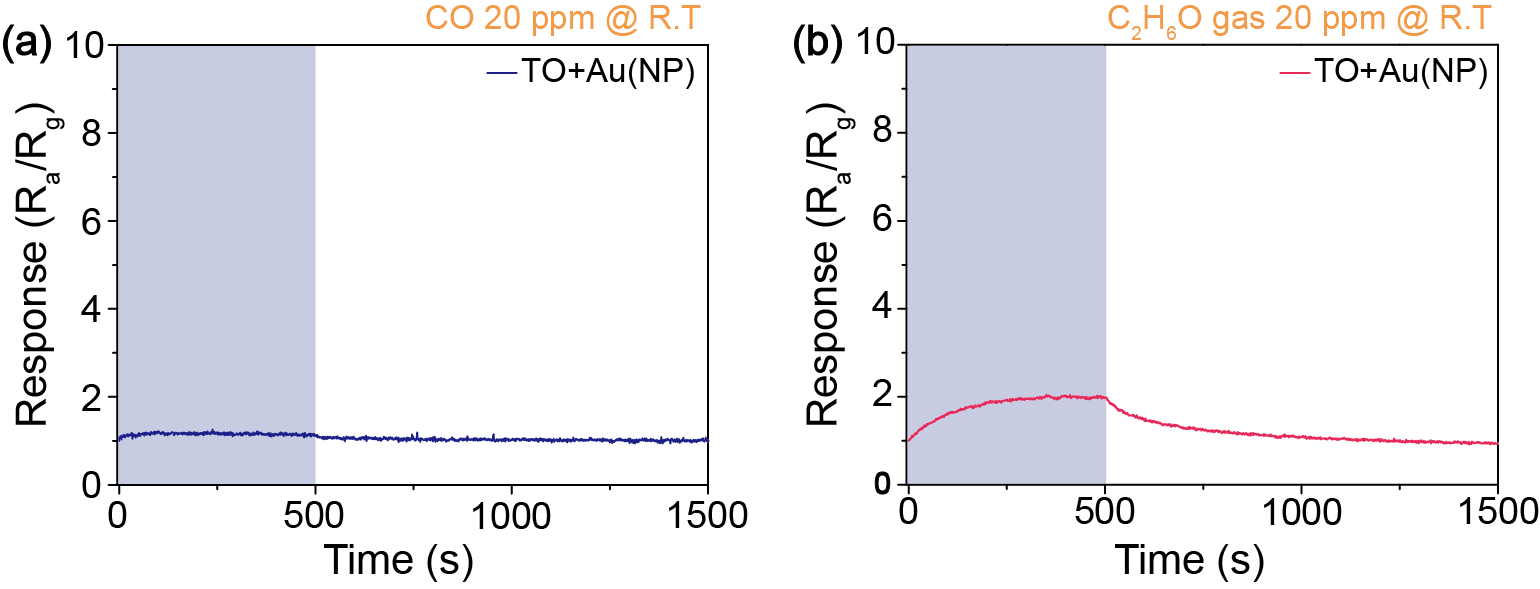


**Figure S13**. Gas sensing response of TO NSs decorated with Au NPs at room temperature. (a) Response to 20 ppm CO and (b) response to 20 ppm C_2_H_6_O.


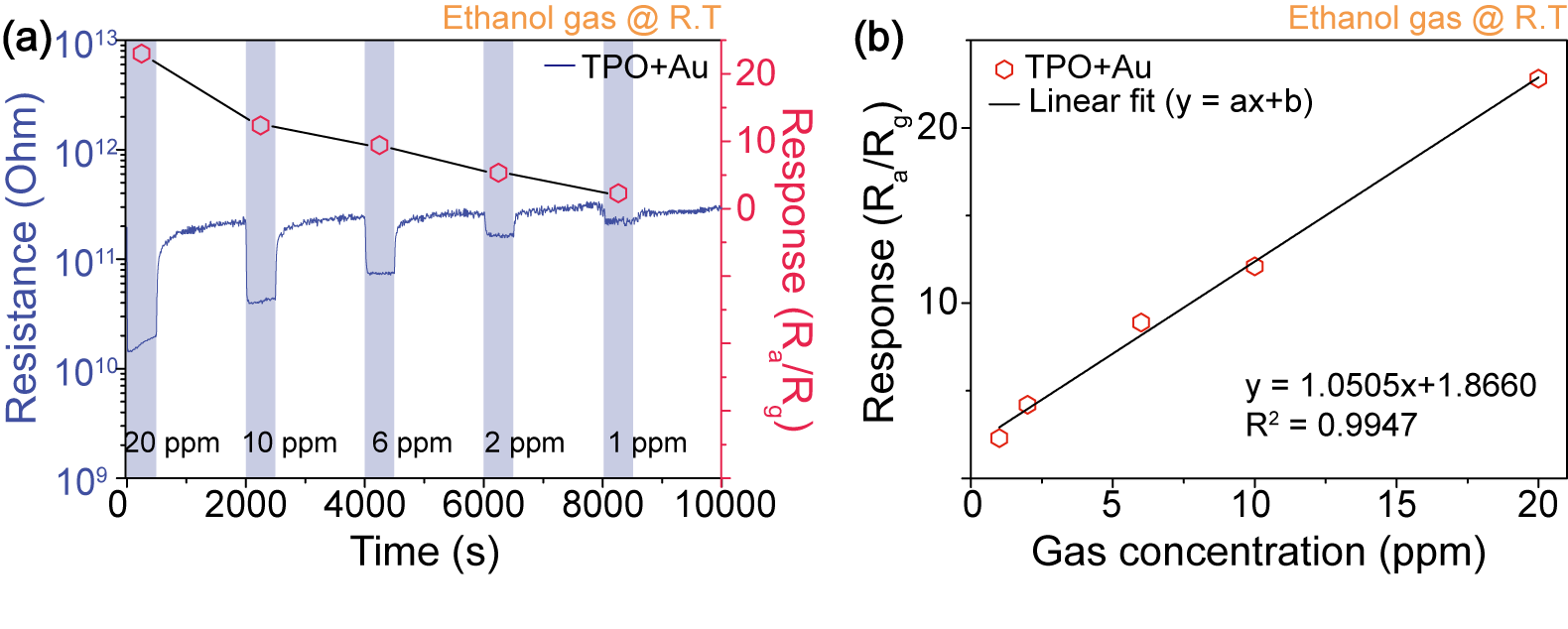


**Figure S14**. Ethanol gas sensing performance of TPO NSs decorated with Au sheets at room temperature. (a) Dynamic resistance and response curves to ethanol concentrations ranging from 1 to 20 ppm and (b) corresponding sensor response versus concentration, indicating the limit of detection (LOD).


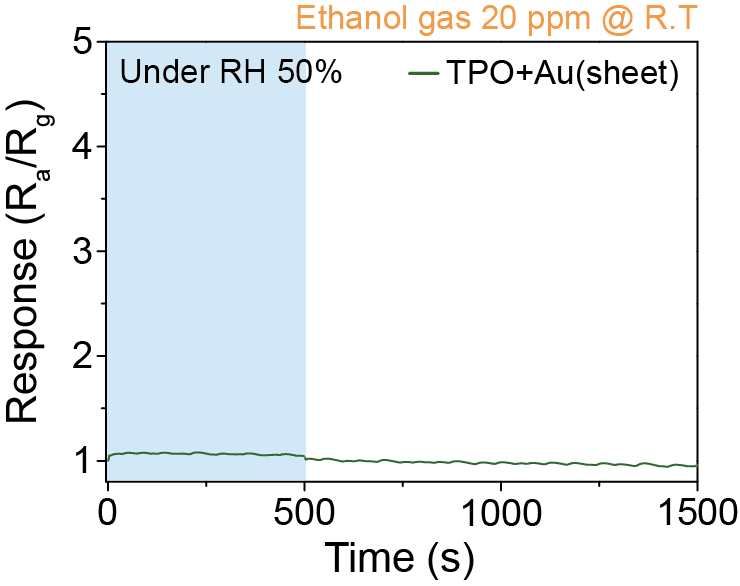


**Figure S15**. Response of TPO NSs decorated with Au sheets to 20 ppm ethanol gas at room temperature under 50 % relative humidity (RH).


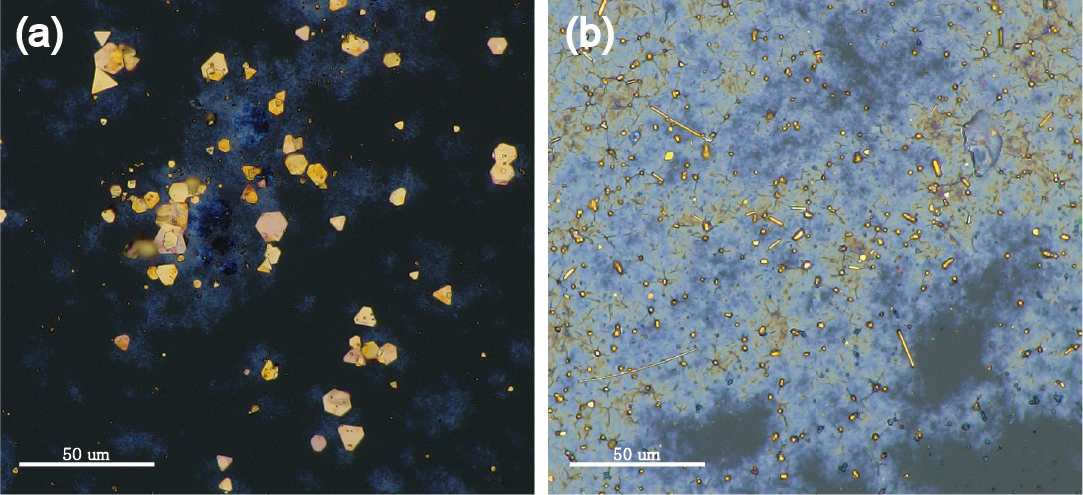


**Figure S16**. OM images of TPO NSs decorated with Au sheets. (a) Before and (b) after Au removal.


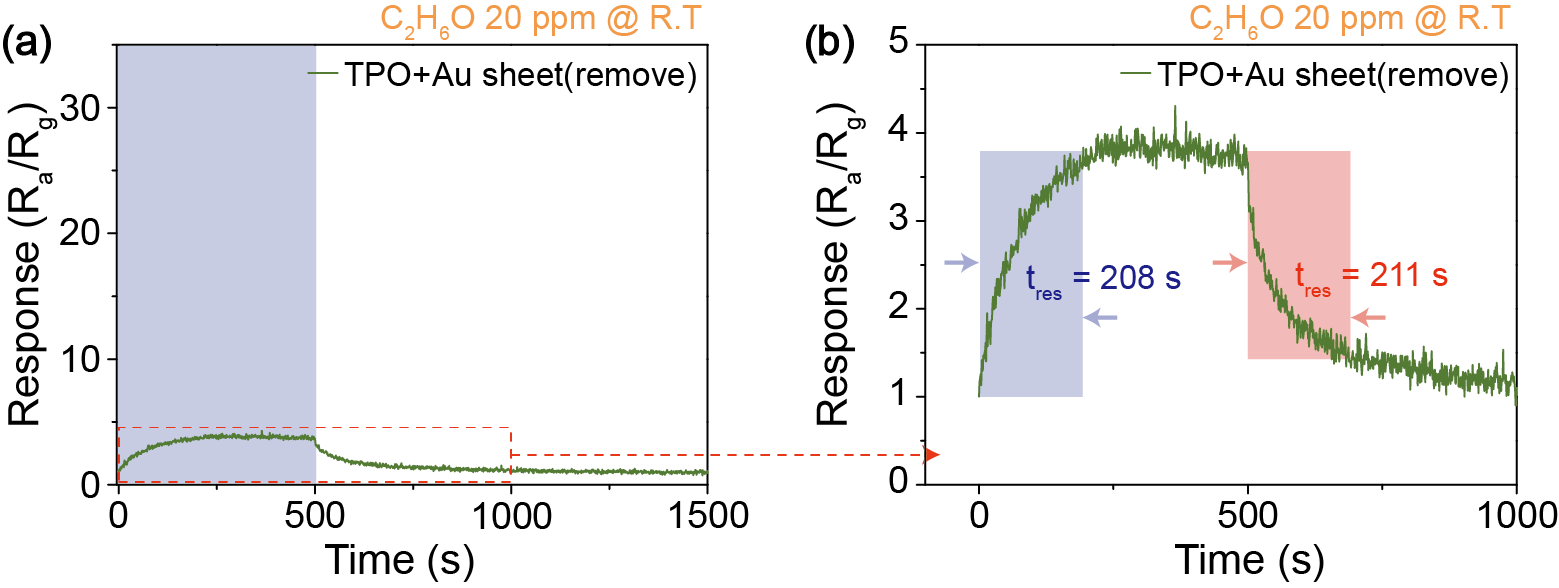


**Figure S17**. Ethanol gas sensing performance of TPO NSs decorated with Au sheets after removal. (a) Dynamic response to 20 ppm ethanol and (b) corresponding response and recovery times.
